# Understanding Biogeochemical Cycling of Trace Elements and Heavy Metals in Estuarine Ecosystems

**DOI:** 10.4172/2155-6199.1000e148

**Published:** 2014

**Authors:** Jacqueline McComb, Turquoise C. Alexander, Fengxiang X. Han, Paul B. Tchounwou

**Affiliations:** 1 Environmental Science Ph.D. Program, Jackson State University, 1400 J.R. Lynch Street, Jackson, MS 39217, USA; 2 Department of Chemistry and Biochemistry, Jackson State University, Jackson, Mississippi, USA

## Editorial

Nature has slow occurring biogeochemical cycles of trace elements and heavy metals and the cycles have significantly controlled environmental fates of these elements. The trace elements and heavy metals cannot be degraded like organic pollutants and they may transform and become stable and persistent contaminants that accumulate in soil and sediments [1–3]. Estuaries play an important role in human civilization such as trading among populations. Many industries are able to take advantage of the estuary for industrial effluents as a source of cold water for heated discharge. As a result, estuaries and coastal marine ecosystems receive a variety of pollutants and contaminants that potentially have adverse effects not only on biota living in these habitats, but also on the humans who consume them [4]. Anthropogenic activities such as pollution, industrial, offshore oil and gas exploration have increased the levels of trace element and metal ions in natural water system. Improper disposal of contaminants from hazardous waste sites and industrial facilities have contributed to the estuary contamination as well. All these toxic elements have been accumulated in soils and sediments and can be ingested or stored in and enter into marina biota (fish and seafood) that is then consumed by humans.

A fundamental understanding of physical behavior of the estuary is essential to clarify the transport of heavy metals and trace elements in an estuary. Estuaries are essentially mixing zones that bring together salt and fresh water and are characterized by their strong chemical and physical gradients. More importantly, salinity is the main factor in the subdivision of contaminants between sediments, overlying, and interstitial waters. The physiochemical parameters of estuaries are more incline to affect the internal processes [5]. For example, processes like precipitation and co-precipitation of solutes and adsorption both aid in the dissolving and removal of trace elements, while resuspension of sediments and solubilisation of particulate matter aid in the production of trace elements in the dissolve phase [6]. Even though trace elements are usually found at concentrations of part per billion, these elements are still significant in estuaries because of their toxicity and the importance as micronutrients for many organisms [7]. In estuaries, the transport of heavy metals and trace elements in soil/sediment is contingent upon the chemical form and speciation of elements. Once they reach the soil/sediments, they are adsorbed on minerals and dispersed as dissimilar chemical forms with varying bioavailability, mobility, and toxicity [8]. Heavy metal and trace element distribution is generally the result of ion exchange, aqueous complexation, biological immobilization, mineral precipitation, and plant uptakes [9].

As a result of the affluence of heavy metals and trace elements, many bio-indicators and eco-indicators have been proposed for enabling detection of heavy-metal and trace element pollution. Oysters are great bioindicators because of their ability to serve as sentinels to monitor the estuary environment, ecological processes, and biodiversity. These changes in the environment can result from human disturbances (e.g. pollution and use changes) and natural stressors (e.g. hurricanes and drought). Barua et al. [10] investigated the seasonal variation of Zn, Cu, Pb, Mn, Ni, Cd, Fe and Co in oysters (*Saccostrea cucullata* ) and water body collected in the northeastern coast of India [10]. Heavy metals accumulated in the water body in the following order: Fe, Mn, Zn, Cu, Pb, Ni, Co, and Cd, while those in oysters: Zn, Fe, Cu, Mn, Pb, Co, Ni, and Cd. Unique seasonal patterns with the highest concentrations during monsoon season and the lowest concentrations during pre-monsoon season. This variation may be attributed to large run-off from adjacent land masses during the monsoon. These results showed that seasonal variations greatly influence trace metals concentration levels during the monsoon season. Changes during seasons, temperature fluctuations in oysters may lead to a bigger concern involving climate change [11]. Relationships of Cu, Pb, Zn, Cd, Cr, Fe and Mn concentrations between sediment and oyster (*Crassostrea virginica* ) tissues and shells have been studies from Gulf of Mexico estuaries [12]. Zinc, cadmium and copper ranked as the top three heavy metals accumulated in the average oyster tissue and oyster shell. Thus, variations in concentrations of heavy metals in different parts of shells can provide a record of environmental changes during oyster growth [9]. We previously reported that native earthworms may be used as a potential mercury ecological bio-indicator (bio-marker) for demonstrating mercury bioavailability and ecotoxicity in the floodplain ecosystem [13]. The results also show strong linear relationships between mercury concentrations in native earthworms (both mature and immature groups) and the non-cinnabar mercury form, while cinnabar mercury is less bioavailable to native earthworms [13].

The subcellular distribution of heavy metals was assayed to better understand the subcellular distribution of metals. Cellular debris was the main subcellular fraction binding the metals. Metallothionein-like proteins increased their importance in binding Zn as tissue concentration of Zn increased [14]. Metal exposure can possibly induce physiological, and biochemical changes in cells receiving inputs of trace metals as a result of the induction of metal detoxification processes. In aquatic invertebrates (e.g. oysters), metals are usually stored in various forms, metal-rich granules (MRG), metallothioneins (MT) or metallothionein-like proteins (MTLP). On the other hand, the enzymatic responses were found in the gills and digestive gland of pearl oyster, *P. fucata* exposed to copper at 0.05 μM and 0.5 μM, respectively [15]. Acid phosphatase, phenoloxidase, superoxide dismutase, Se-dependent glutathione peroxidase and alkaline phosphatase were five enzymes that were investigated. The results supported the proper usage of enzymes as biomarkers and laid a foundation for understanding of the defense mechanism of the pearl oyster [15].

Marine organisms such as oysters have the ability to concentrate high amounts of arsenic in their tissues [16]. The ubiquitous element arsenobetaine in marine animals has been studied for many years [17]. The results showed arsenic concentration of each tissue part ranged from 22.1 to 45.7 μg g^−1^ of dry tissues in the pearl free oysters and from 27.4 to 50.4 μg g^−1^ of dry tissue in the pearl-containing pearl oysters [16].

Zinc is another common essential trace metal element found in the Earth’s crust, in the air, soil, water, and is present in all foods [18]. Zinc compounds are commonly used in the pharmaceuticals industry as ingredients in some common products, such as vitamin supplements, sun blocks, diaper rash ointments, deodorants and antidandruff shampoos [18]. Zinc is an essential trace metal with biological and public health significance. An inadequate amount of zinc has been associated with growth retardation, delayed maturation and infection susceptibility [19]. Zinc homeostasis is primarily maintained through the gastrointestinal system (small intestine, liver and pancreas) via absorption of exogenous zinc and gastrointestinal secretion and excretion of endogenous zinc [20]. Zinc functions as a cofactor for many enzymes in both aquatic and terrestrial organisms [21]. Oysters are a good source of Zn and are considered an important dietary Zn supplement [22]. Zinc is regulated in marine organisms; however oysters tend to accumulate Zn to a very high body concentration [23].

Zn exposure could affect the overall bioaccumulation of Cd and Cu in three populations of the oyster *Crassostrea hongkongensis* [24]. The results suggest that the increased bioaccumulation of Cd and Cu is highly dependent on tissue Zn concentration. The magnitude from high bioaccumulation levels is most likely associated with a history of metal pre-exposure. The results revealed that as the tissue Zn concentration increased, the concentration of Cd/Cu associated with metallothionein-like proteins (MTLP), metal-rich granules (MRG) progressively increased [24].

Transition metals (e.g. zinc and cadmium) act as catalysts in the oxidative reactions in biological macromolecules. Therefore, metal toxicity may be associated with oxidative tissue damage [25]. Organisms guard themselves from metal-induced damage at the molecular and cellular levels using two protective strategies. First, the cellular defense aids to repair and protect macromolecules from metal toxicity. Second, the metal-specific induction of metallothioneins (MTs) synthesis is used to change metal metabolism. MT is a collective name for a superfamily of low molecular weight (6–7 kDa) metal-binding proteins or polypeptides with extremely high thiolate sulfur and metal content [24]. MTs are likely to be involved in zinc metabolism and protection against certain metal toxicities [26]. Metallothioneins (MTs) or MTLP are recognized in the detoxification of accumulated metals, although the biological significance and reactivity of the different detoxified forms remain unknown. MTLP induction is one of the well-documented strategies in metal detoxification in aquatic animals, and the metal-binding proteins can sequestrate excess intracellular metals effectively. MTLP is important because it serves as protection from metal toxicity [27,28]. Despite several studies on Zn uptake and depuration in oysters, the exact mechanism of the regulation of Zn tissue concentration is not understood.

Scientific literature revealed that the majority of experimental studies on accumulation of trace elements and heavy metals in oysters were based on dose-response experiments. In these experiments, the exposure levels were usually higher than the real environmental conditions where the marine organisms were located [29]. The high exposure levels of trace metals in oysters allowed measuring bioaccumulation and controlling exposure levels in seawater while detecting ecotoxicological responses to contamination using current applied analytical techniques. According to a study conducted by Sanudo-Wilhelmy [30], approximately 70% of articles about dissolved metals in National Oceanic and Atmospheric Administration (NOAA) and in the United States Environment Protection Agency (U.S. EPA) programs are from five estuaries: Rhode Island, New York, South Carolina, Delaware, and Maryland in the United States [30].

There is an increasing interest in biogeochemistry of trace elements and heavy metals in marine ecosystems, due to the rapid and observable changes [31–35]. Comparative approach (observational and conceptual model) should provide a deep insight for the study of ecosystem responses to anthropogenic pollution and biogeochemical responses. Although heavy metal and trace element contamination in estuary ecosystem remains evident, even more background knowledge of sources, chemistry, toxicity, in contaminated soils is needed to provide an alternate for detecting the sensitivity of estuarine ecosystems [32].
